# Rapid diagnostic tests as a source of DNA for *Plasmodium *species-specific real-time PCR

**DOI:** 10.1186/1475-2875-10-67

**Published:** 2011-03-24

**Authors:** Lieselotte Cnops, Merel Boderie, Philippe Gillet, Marjan Van Esbroeck, Jan Jacobs

**Affiliations:** 1 Department of Clinical Sciences, Institute of Tropical Medicine (ITM), Antwerp, Belgium; 2 Medical Microbiology, Faculty of Health, Medicine and Life Sciences (FHML), Maastricht, The Netherlands

## Abstract

**Background:**

This study describes the use of malaria rapid diagnostic tests (RDTs) as a source of DNA for *Plasmodium *species-specific real-time PCR.

**Methods:**

First, the best method to recover DNA from RDTs was investigated and then the applicability of this DNA extraction method was assessed on 12 different RDT brands. Finally, two RDT brands (OptiMAL Rapid Malaria Test and SDFK60 malaria Ag *Plasmodium falciparum*/Pan test) were comprehensively evaluated on a panel of clinical samples submitted for routine malaria diagnosis at ITM. DNA amplification was done with the 18S rRNA real-time PCR targeting the four *Plasmodium *species. Results of PCR on RDT were compared to those obtained by PCR on whole blood samples.

**Results:**

Best results were obtained by isolating DNA from the proximal part of the nitrocellulose component of the RDT strip with a simple DNA elution method. The PCR on RDT showed a detection limit of 0.02 asexual parasites/μl, which was identical to the same PCR on whole blood. For all 12 RDT brands tested, DNA was detected except for one brand when a low parasite density sample was applied. In RDTs with a plastic seal covering the nitrocellulose strip, DNA extraction was hampered. PCR analysis on clinical RDT samples demonstrated correct identification for single species infections for all RDT samples with asexual parasites of *P. falciparum *(n = 60), *Plasmodium vivax *(n = 10), *Plasmodium ovale *(n = 10) and *Plasmodium malariae *(n = 10). Samples with only gametocytes were detected in all OptiMAL and in 10 of the 11 SDFK60 tests. None of the negative samples (n = 20) gave a signal by PCR on RDT. With PCR on RDT, higher Ct-values were observed than with PCR on whole blood, with a mean difference of 2.68 for OptiMAL and 3.53 for SDFK60. Mixed infections were correctly identified with PCR on RDT in 4/5 OptiMAL tests and 2/5 SDFK60 tests.

**Conclusions:**

RDTs are a reliable source of DNA for *Plasmodium *real-time PCR. This study demonstrates the best method of RDT fragment sampling for a wide range of RDT brands in combination with a simple and low cost extraction method, allowing RDT quality control.

## Background

Rapid diagnostic tests (RDTs) are frequently used as an adjunct to microscopy in the diagnosis of malaria [1] and even as a point-of-care diagnostic tool [2]. In settings where high quality microscopy is not available, the detection of *Plasmodium *infections is often based on RDTs alone [3,4]. World Health Organization (WHO) recommends the use of RDTs as part of parasite-based diagnosis and supports the broad implementation of RDTs for malaria diagnosis in areas where malaria is prevalent [5-7]. Although fast and simple in concept, RDT performance in practice requires well-trained operators that are able to interpret results correctly and record them properly. At present, there is no widely accepted way of assessing the quality of RDTs at the end-user level and both microscopy and PCR could be used as reference method [8].

Recently, a species-specific *Plasmodium *real-time PCR was successfully applied on stained thick blood films as the source of DNA. Such PCR on slides can have applications in clinical and research settings in case whole blood samples are not available [9,10]. Likewise, PCR applied on RDTs would be beneficial, for instance as quality control of RDTs used in endemic settings. In addition, the use of stored RDTs as source of DNA for PCR amplification might obviate the need for collection of whole blood or filter-based blood samples.

The success of the PCR depends in particular on the accurate extraction of high quality DNA. Therefore, in this study, different RDT components were firstly evaluated as a source to recover *Plasmodium *DNA by real-time PCR. The best sampling and DNA extraction methods were explored. Secondly, the applicability of this method was tested on a range of twelve different RDT brands. Thirdly, the accuracy of PCR on RDT was fully evaluated by challenging it with a panel of clinical samples comprising the four *Plasmodium *species at different parasite densities.

## Methods

### Laboratory diagnosis of malaria at ITM

Clinical samples were obtained from patients suspected of malaria presenting at the outpatient clinic of the Institute of Tropical Medicine (ITM) Antwerp, Belgium or were submitted by Belgian laboratories to the Central Laboratory of Clinical Biology of ITM in the scope of its national reference function for the diagnosis of *Plasmodium*. Malaria diagnosis at ITM is accredited according to ISO 15189:2007 and is done by the combination of standard microscopy, antigen detection and real-time PCR.

Giemsa-stained thick blood films were examined by light microscopy using a × 1000 magnification. Parasite density was determined as described before [11] and expressed as the number of asexual parasites per microlitre (/μl). Species identification was done by microscopy on May-Grünwald Giemsa-stained thin blood films.

Antigen detection was performed by two RDTs: 1) the SD-FK60 Malaria Ag Pf/pan test (Standard Diagnostics, Hagal-Dong, Korea, further referred to as SDFK60) detecting *P. falciparum *(Pf) histidine-rich protein-2 (HRP-2) and pan-species parasite lactate dehydrogenase (pLDH), and, 2) the OptiMAL^® ^pLDH (Pan, Pf) (Diamed AG, Cressier, Switzerland, further referred to as OptiMAL) targeting Pf-specific pLDH and pan-species specific pLDH.

All samples that were positive with microscopy or antigen testing were analyzed by real-time PCR on 200 μl of fresh EDTA-anticoagulated blood for confirmation or correction of the species as diagnosed by microscopy [9]. In case of discordant results between microscopy and PCR, the results of PCR on whole blood were considered as the reference method. Laboratory diagnosis for malaria was considered negative when both microscopy and PCR were negative.

### Construction and design of the RDT device

**Figure 1:**
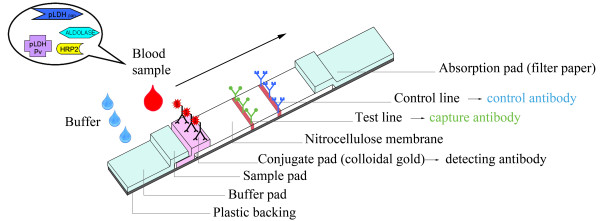
**Different components of a two-band RDT**. Blood is applied on the sample pad at the proximal part of the RDT and buffer is dropped on the buffer pad. The absorption pad that consists of filter paper sucks the solution to the distal part of the RDT. Detecting antibodies conjugated with colloid gold are present on the conjugate pad coloring it pink. On the nitrocellulose membrane, test lines with capture antibodies for HRP-2, pLDH or aldolase and a control line with control antibodies are present. Depending on the RDT brand, the sample pad, buffer pad and conjugate pad are either separate or combined in one component. Those three pads consist of fiberglass-like paper strips and partly overlap the nitrocellulose membrane. All components are glued onto a plastic backing. Some RDTs contain a plastic cover on top of the nitrocellulose membrane (not illustrated). Most RDTs are packed in a plastic cassette housing or available as cardboard or hybrid format. The arrow indicates the direction of the lateral flow from proximal to distal RDT components.

Figure 1 shows the different components on a two-band RDT strip. The strip consists of a plastic backing on which several components are mounted; these components partly overlap each other. The buffer, sample and conjugate pads are located at the proximal part of the RDT device. In the middle, a nitrocellulose membrane is fixed on the backing. The distal part contains a filter paper component that functions as absorption pad. In most cases, the strip with its components is embedded in a plastic cassette.

The sequence of events of a RDT test is as follows: the blood sample is applied to the sample pad after which RDT buffer is dropped onto the buffer pad. The buffer and the blood mix with each other and the solution migrates towards the distal part of the RDT strip, driven by capillary forces of the filter paper at the absorption pad. During the migration, the solution passes the conjugate pad, which contains detecting mouse antibody targeting a *Plasmodium *antigen such as HRP-2, Pf-pLDH, pan-pLDH or aldolase. These detecting antibodies are labeled (conjugated) with a signal, mostly colloidal gold particles. If present in the sample, the *Plasmodium *antigen binds to the detecting antibody-conjugate. The antigen-antibody-conjugate complex then further migrates across the nitrocellulose membrane towards the absorption pad until it binds to capture antibodies that are applied on a narrow section of the nitrocellulose membrane. By binding to the capture antibodies, the antigen-antibody-conjugate complex is concentrated onto the small section and becomes visible as a cherry-red coloured test line. The excess of detection antibody-conjugate that is not bound by antigens moves further until it binds to goat-raised anti-mouse control antibodies, thereby generating a control line. The residual blood solution is absorbed by the filter paper of the absorption pad.

### Panel of different RDT brands assessed

Twelve RDT brands were evaluated. The selection of RDTs was based on their use in Belgian laboratories [12] and in endemic settings [13]. They represented different formats (two-band and three-band), devices, target antigens, as well as different designs (with or without plastic seal covering the nitrocellulose strip) (Table 1). Ten out of twelve RDTs were cassettes, one was a cardboard device and another was a hybrid strip device.

**Table 1:** Panel of twelve RDT brands selected for evaluation of the applicability of the RDT fragment sampling and DNA extraction method

RDT name	Manufacturer	Format	Device	Target antigens
**OptiMAL-IT**	DiaMed AG, Cressier, Switzerland	3-band	hybrid	pan-pLDH Pf-pLDH
**SD Bioline Malaria Ag Pf FK50**	Standard diagnostics Inc, Hagal-Dong, Korea	2-band	cassette	HRP-2
**SD Bioline Malaria Ag Pf/Pan FK60**	Standard diagnostics Inc, Hagal-Dong, Korea	3-band	cassette	HRP-2 pan-pLDH
**Advantage Pan MAL card**	J. Miltra & co. Pvt. Ltd. New Delhi, India	2-band	cassette	Pan-pLDH
First response^® ^Malaria pLDH/HRP2 Combo test*	Premier Medical Corporation Ltd, Daman, India	3-band	cassette	HRP-2 pan-pLDH
**ICT Malaria Pf**	ICT diagnostics, Cape Town, South Africa	2-band	cassette	HRP-2
**ParaHIT total**	Span Diagnostics Ltd, Sachin, India	3-band	cassette	HRP-2 aldolose/pan pLDH
**BinaxNOW^® ^Malaria test**	Inverness Medical Binax, Inc Sacrborough, Maine, USA	3-band	cardboard	HRP-2 aldolase
**Core**^**TM **^Malaria PAN/Pf *	Core Diagnostics, Birmingham, UK	3-band	cassette	HRP-2 pan-pLDH
**Hexagon Malaria Combi**	Human, Wiesbaden, Germany	3-band	cassette	HRP-2 aldolase
Paracheck Pf*	Orchid Biomedical Systems, Goa, India	2-band	cassette	HRP-2
**Carestart**^**TM**^Malaria HRP2 test*	Access Bio Inc, New Jersey, USA	2-band	cassette	HRP-2

The underlined names represent the shortened names further used in the text

* RDT with plastic seal covering the nitrocellulose membrane

### Selection of the most appropriate RDT fragment

Each component of the RDT strip that makes contact with the blood sample during the antigen detection process was considered and assessed as a possible carrier of parasite DNA (Figure 1). For each RDT brand, a fragment of the different RDT components was evaluated as specimen for DNA extraction (Table 2 and 3).

**Table 2:** Optimization of RDT fragment sampling and DNA extraction method

1) Selection of RDT fragment			
	Proximal nitrocellulose	Distal nitrocellulose	Filter paper
	
**OptiMal **(Qiagen method)	**27.57**	0	39.09
	Proximal nitrocellulose	Conjugate pad	Filter paper
	
**SDFK60 **(Qiagen method)	30.55*	38.62	38.62
			
**2) Selection of DNA extraction method**			

	Elution method	Qiagen method	
	
**OptiMal **(nitrocellulose strip)	**28.59**	32.64	
**SDFK60 **(nitrocellulose strip)	**31.53**	36.12	

Cycle threshold (Ct)-values in bold indicate best PCR results (i.e. lowest Ct-values)

* fragment sampling of the nitrocellulose strip together with the conjugate pad gave a Ct-value of 29.28

**Table 3:** Evaluation of PCR on RDT on 12 RDT brands

		Results for P. falciparum with 21/μl PCR on whole blood Ct 33.52		Results for P. falciparum with 830/μl PCR on whole blood Ct 26.86		
		
RDT name	blood volume	Ag detection	DNA detection (Ct)	Ag detection	DNA detection (Ct)	RDT fragment used
**OptiMAL**	10 μl	n	34,67	p	30,12	1/3 NC
**SDFK50**	5 μl	p	35,32	p	30,36	1/2 NC + C
**SDFK60**	5 μl	p	35,47	p	30,20	1/2 NC + C
**Pan MAL card**	5 μl	n	35,77	p	30,49	1/2 NC
First response*	5 μl	p	35,88	p	31,48	2/3 S/C
**ICT**	5 μl	p	36,74	p	30,18	1/2 NC
**ParaHIT**	8 μl	p	37,56	p	33,20	1/2 C
**Binax**	15 μl	p	37,77	p	30,19	1/2 NC
Core*	5 μl	p	38,82	p	32,83	2/3 S/C
**Hexagon**	5 μl	p	39,58	p	34,08	1/2 NC
Paracheck*	5 μl	p	40,53**	p	37,33	C
Carestart*	5 μl	p	0	p	39,21	2/3 S/C

-Antigen detection is considered positive (p) as test line(s) was (were) visible and negative (n) if no test line was visible.

-DNA detection by real-time PCR on RDT is expressed in Cycle threshold (Ct)-values and RDTs were ranked in order of increasing Ct-values.

**Figure 2:**
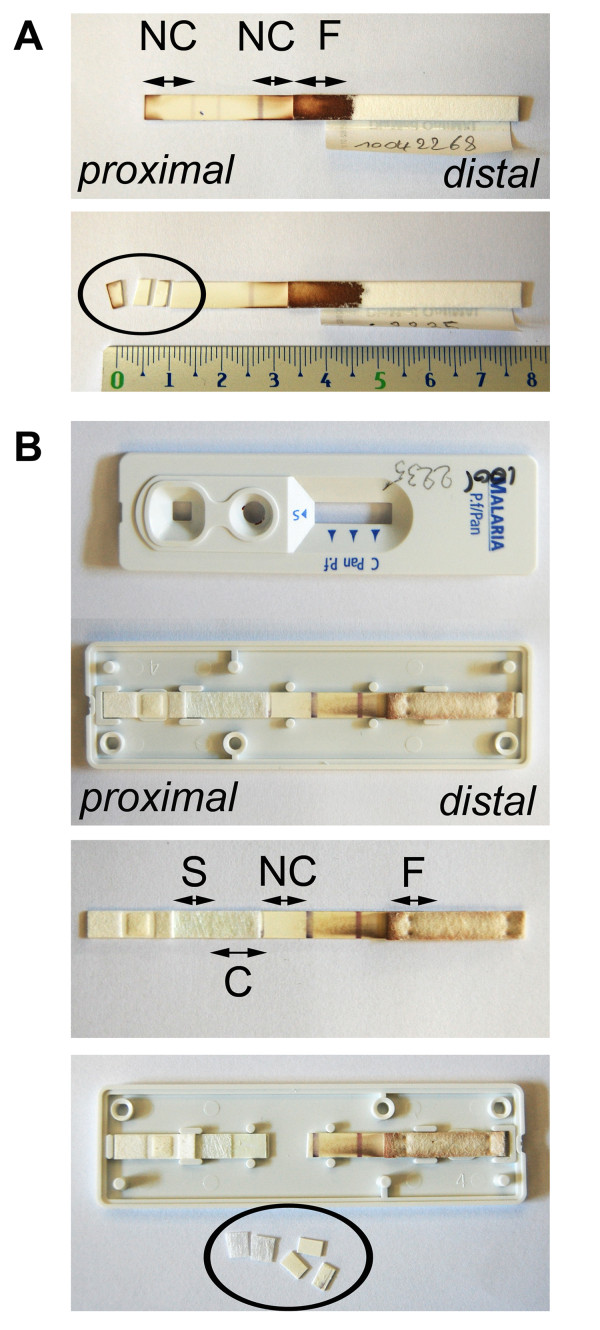
**Fragment sampling of OptiMAL (A) and SDFK60 (B)**. NC = nitrocellulose strip (nitrocellulose membrane inclusive plastic backing), F = filter paper fragment of absorption pad, S = sample pad, C = conjugate pad.

-RDT fragments used were either one third (1/3) or half (1/2) of the proximal part of the nitrocellulose (NC) strip or (two third of) the sample (S) and/or conjugate (C) pad (see Figure 2).

* RDTs with a plastic seal covering ik the nitrocellulose strip

** In the first test, no signal was seen (Ct-value 0), only by repetition of the DNA extraction a positive signal (Ct-value 40.53) was observed.

For the purpose of this experiment, RDTs of each brand were seeded with two blood samples that contained *P. falciparum *at parasite densities of 21/μl and 830/μl respectively. The corresponding Ct-values of the PCR applied on whole blood were 33.52 and 26.86 respectively. The blood volume used for each RDT was as indicated by the manufacturer (Table 3) and applied by a transfer pipette (Finnpippette, Helsinki, Finland). RDTs were processed according to the instructions of the manufacturer and test results were read after the recommended reading time by two observers. After at least one week of storage at room temperature, fragments of the following components (if present) were sampled and assessed apart or combined: buffer pad, sample pad, conjugate pad, nitrocellulose strip, filter paper and plastic seal (Figure 1). Fragments were cut with a sterile scalpel (number 15, Farla Medicals, Antwerp, Belgium) in two or three pieces of about 2 mm. For each RDT a separate scalpel was used. The fibreglass-like paper strips of the buffer pad, sample pad and/or the conjugate pad were easily lifted up from the underlying backing. By contrast, the nitrocellulose membrane is tightly glued to the backing. Unless stated otherwise, the fragments of the nitrocellulose membrane were sampled together with this backing and are further referred to as the nitrocellulose strip. From the absorption pad, the proximal part of the filter paper without backing was used. In four brands, a plastic seal was covering the nitrocellulose membrane; when peeling off, the nitrocellulose membrane remained partly stuck to this plastic seal, making preparation of the entire nitrocellulose strip difficult.

### Panel of clinical samples used for evaluation of the PCR on RDT

To evaluate the performance of the PCR on RDT on clinical samples, a panel of SDFK60 and OptiMAL RDTs used as part of routine laboratory work-up during the period February 2009 and April 2010 was selected. After reading the RDT test result, SDFK60 and OptiMAL RDT devices were individually stored without silica-gel in a dry and dark closet at ambient temperature below 25°C until PCR analysis was performed. The RDTs represented single infections by the four *Plasmodium *species at different parasite densities (ranging from 1 to 222.241/μl) or with only gametocytes, malaria negative samples and mixed infections. In addition, RDTs performed on samples of patients under malaria treatment were included: these samples were negative by microscopy, but *P. falciparum *DNA and HRP-2 antigens were still detected by PCR on whole blood and SDFK60 respectively.

### Fragment sampling for OptiMAL and SDFK60 RDTs of clinical samples

For the OptiMAL, the proximal 1/3 part of the nitrocellulose strip just before the first test line was cut in three pieces of about 2 mm (Figure 2A). For the SDFK60, the cassette was opened laterally and the strip was taken out. The conjugate pad was lifted up from the backing and cut in two pieces of about 3 mm. In addition, three pieces of 2 mm of the proximal part of the nitrocellulose strip until the first test line were sampled (Figure 2B). Between each RDT fragment sampling, the working area was cleaned with 0.5% hypochlorite solution and 70% ethanol.

### DNA extraction from RDT fragments

During optimization, DNA from OptiMAL and SDFK60 fragments was extracted by the QIAamp DNA mini kit (Qiagen Benelux, Venlo, The Netherlands) as described before [9] and by a simple elution method in water that was adapted from Volpini *et al *[14]. Because of better DNA recovery (see Results section), the latter method was selected for all further experiments and all RDT brands. For DNA elution, fragments were placed in a 1.5 ml tube containing 50 μl molecular grade water (VWR, Leuven, Belgium). Samples were thoroughly mixed by vortex for 10 seconds. After briefly centrifuging to remove drops from the inside of the lid and to make sure all fragment were below the water surface, samples were heated at 95°C for 10 minutes. Hereafter, tubes were centrifuged for 5 minutes at 8000 × g and the supernatant was transferred to a new tube of which 5 μl was used for PCR. Within each batch of RDT extractions, at least one RDT of a *Plasmodium *negative sample was included and randomly ranked among the positive RDTs. Efficient extraction was controlled by the detection of human DNA with the human-beta-globin (HBB) PCR [10].

### Real-time PCR on RDT

The 'four-primer' real-time PCR with a non-competitive design was used as described previously [9]. Shortly, four *Plasmodium *species-specific forward primers and four *Plasmodium *species-specific probes together with one *Plasmodium *genus-specific reverse primer were used to target the 18S small subunit rRNA gene of the four *Plasmodium *species. Two duplex reactions, one to detect *P. falciparum *and *Plasmodium vivax *and another to detect *Plasmodium ovale *and *Plasmodium malariae*, were run in parallel for 2 min at 95°C followed by 50 cycles of 15 sec at 95°C and 60 sec at 60°C on the SmartCycler II (Cepheid Benelux, Bouwel, Belgium).

### Analytical sensitivity

To determine the analytical sensitivity, serial ten-fold dilutions were made from a single clinical blood sample infected with *P. falciparum *at a parasite density of 206.100/μl. OptiMAL and SDFK60 RDTs were seeded with each dilution and DNA was eluted as described above. PCR was simultaneously performed on all dilutions. The highest dilution with a positive PCR signal indicated the detection limit.

### Reproducibility

To determine the reproducibility of the DNA extraction from RDTs by PCR, four OptiMAL and four SDFK60 RDTs were seeded with a single *P. falciparum*-positive sample with 1.110/μl and DNA was extracted from each RDT. The subsequent PCR was performed in four separate PCR runs and the coefficient of variation (%CV) of the Ct-values was calculated.

### Data analysis

PCR results were expressed as cycle threshold (Ct)-values and low Ct-values correspond to high DNA levels. Negative samples generate no PCR signal (Ct-value = 0). The correlation between Ct-values obtained by PCR on whole blood and PCR on RDT was determined by linear regression analysis with the Pearson correlation coefficient indicated by R^2^. Statistical differences between logarithmic Ct-values obtained by PCR on whole blood and PCR on RDT were determined by paired *t*-test analysis and defined as statistically significant different if p < 0.01.

The Ct-values measured by PCR on RDT and PCR on whole blood were subtracted (indicated by ΔCt). The mean of all ΔCt-values was calculated together with the ± 95% confidence interval of the mean ΔCt. Theoretically, the amount of amplicon doubles (*i.e. *increase by one log_2_) every cycle and one ΔCt and three ΔCt's corresponds thus respectively to a two-fold and eight-fold increase.

## Results

### Optimization of the RDT fragment sampling and DNA extraction method

In a first set of experiments, DNA from three RDT fragments was extracted by the Qiagen method. For the OptiMAL, the nitrocellulose strip (proximal and distal parts) and the filter paper (proximal part) were assessed (Figure 2A). For the SDFK60, the nitrocellulose strip, the conjugate pad and the filter paper (proximal part) were used (Figure 2B). For both RDTs, the proximal part of the nitrocellulose strip generated the best result (lowest Ct-value) (Table 2).

In a second experiment, the nitrocellulose strip of both RDT brands was used to compare the Qiagen extraction method to the elution method. Best results were obtained by the elution method with PCR signals that were 4 to 5 Ct-values lower than the Qiagen method. Further experiments with the SDFK60 demonstrated that samples consisting of the combination of the proximal part of the nitrocellulose strip and the conjugate pad generated lower Ct-values as compared to the fragments that were processed apart (Table 2).

All following experiments were performed with the proximal part of the nitrocellulose strip of the OptiMAL (Figure 2A, lower panel) and the conjugate pad together with the proximal part of the nitrocellulose strip of the SDFK60 (Figure 2B, lower panel). HBB PCR could not be used as extraction control as no or very weak HBB signals were seen in DNA extracts from RDTs.

During the optimization phase, fragments were cut on a Harris cutting mat (Whatman, Kent, UK). Once, a contamination with *P. falciparum *was noticed in a negative RDT sample by the preceding RDT that was extracted in the same batch. For all further experiments, it was decided to sample the fragments of each RDT device on a new tissue-paper.

### Applicability of the fragment sampling and DNA elution method on twelve RDT brands

The elution method was applied on 12 RDT brands seeded with two clinical samples of *P. falciparum *at parasite densities of 21/μl and 830/μl. To select the most appropriate RDT fragment for each RDT brand, a single fragment or the combination of fragments were tested with the DNA elution method. Based on the above-described results, selection of fragments was focused on the nitrocellulose strip, sample pad and conjugate pad.

Table 3 lists the twelve RDT brands with the Ct-values as observed for the fragment(s) that obtained best Ct-values upon elution and PCR. Depending on the design of the RDT device, selection of the most appropriate fragment differed. For seven RDTs, DNA was recovered from the nitrocellulose strip. For Binax, half of the nitrocellulose membrane was scraped off from the backing, and the latter was not included for DNA extraction. This manner of fragment sampling revealed a better Ct-value compared to the nitrocellulose strip and was easier to perform as the plastic backing and the housing cardboard were tightly glued to each other. For RDTs with a plastic cover on top of nitrocellulose strip (indicated by * in Table 3), the recovery of DNA was hampered as the nitrocellulose membrane remained partly fixed to the plastic cover while removing the cover. Isolation of DNA from the plastic cover revealed very high Ct-values or no PCR signal. For that reason, only the sample/conjugate pad or conjugate pad was used for DNA extraction for RDTs with a plastic cover. For the ParaHIT, which has a sample/buffer pad that completely covers the conjugate pad, no signal was detected for the nitrocellulose strip, and the best Ct-values were seen for the distal part of the conjugate pad.

DNA amplification succeeded for all RDT brands seeded with the *P. falciparum *sample of 830/μl and all but one RDT brands seeded with the *P. falciparum *sample of 21/μl. Based on comparison of the Ct-values, best PCR results were observed for the OptiMAL test. Two different brands of the same company (SDFK50 and SDFK60) performed equally well and weakest results were generated for the Hexagon, Paracheck and Carestart (Table 3). In two cases, no antigen test line was observed while PCR on RDT was positive. There was no relation between the blood volume applied for the different RDT brands and the corresponding Ct-value: for instance, in line with the manufacturer's instructions, a three times higher blood volume was applied on the Binax RDT as compared to the SDFK brands (15 μl versus 5 μl), but the recovery of parasite DNA from the Binax RDT was lower than from the SDFK brands for the low parasite density sample.

### Analytical sensitivity and reproducibility of PCR results

The analytical sensitivity test on serial dilutions of the blood sample containing *P. falciparum *demonstrated a detection limit of 0.02/μl for OptiMAL and SDFK60 which respectively corresponds to 0.2 and 0.1 asexual parasites per unit of blood volume applied to each RDT. Reproducibility testing for OptiMAL and SDFK60 RDTs showed a CV of 0.8% and 3.1% respectively.

### Analysis of clinical samples by PCR on RDT

PCR was performed between May and august 2010 on OptiMAL and SDFK60 RDTs from 121 clinical samples (Table 4) that were stored for at least one week up to 16 months with a median storage time of 10 months. PCR on OptiMAL showed correct species identification for all 91 samples containing a single *Plasmodium *species, even so for samples with low parasite densities (< 100/μl) and only gametocytes. With the PCR on SDFK60, 90/91 samples were correctly detected; a single *P. falciparum *sample with only gametocytes was missed.

**Table 4:** Results of PCR on RDT and presence of antigen test lines for OptiMAL and SDFK60 tested with a panel of clinical samples

			OptiMAL		SDFK60	
			
			Correct DNA detection by PCR on RDT	Correct antigen detection by RDT	Correct DNA detection by PCR on RDT	Correct antigen detection by RDT
			
PCR on whole blood	Parasite density	Nrs	Nrs	Nrs	Nrs	Nrs
*P. falciparum*	only gametocytes	**10**	**10**	6	**9**	9
	1-100	**10**	**10**	4	**10**	10
	101-500	**15**	**15**	14	**15**	15
	501-1000	**5**	**5**	5	**5**	5
	>1000	**20**	**20**	20	**20**	20
*P. vivax*	only gametocytes	**1**	**1**	0	**1**	0
	1 to >1000	**10**	**10**	10	**10**	9
*P. ovale*	1 to >1000	**10**	**10**	2	**10**	4
*P. malariae*	1 to >1000	**10**	**10**	6	**10**	6

Total single infections	0 to >1000	**91**	**91**	67	**90**	78
						
Mixed infections	1 to >1000	**5**	**4**	3^#^	**2**	1^#^
After start of treatment	0	**15**	**11**	1	**7**	15
Negative	0	**10**	**10**	10	**10**	10

**Total**		**121**	**116**	**81**	**109**	**104**

PCR on whole blood was the reference method.

# numbers of RDT samples with two positive test lines

Mixed infections were detected by PCR in 4/5 and 2/5 of the RDT samples for OptiMAL and SDFK60 respectively (Table 5). The mixed infection that was missed by PCR on OptiMAL was detected by PCR on SDFK60 and *vice versa*. In the other missed mixed infections, only the major species was detected by PCR on RDT (Table 5).

**Table 5:** Detection of mixed infection by PCR on whole blood, PCR on RDT and antigen detection.

		OptiMAL		SDFK60	
		
	PCR on whole blood	PCR on RDT	Antigen detection	PCR on RDT	Antigen detection
	
Mixed infection	Ct-value	Ct-value	Test line(s)	Ct-value	Test line(s)
*P. falciparum +P. malariae*	36.06 + **26.50**	38.23 + **30.13**	Pan pLDH	0.00 + **31.17**	negative
*P. falciparum +P. malariae*	**29.68 **+33.63	**32.33 **+ 39.12	Pf + pan pLDH	**34.46 **+ 37.93	HRP-2
*P. falciparum +P. malariae*	**25.88 **+ 37.06	**27.77 **+ 39.65	Pfmx	**29.97 **+ 0.00	HRP-2 + pLDH
*P. falciparum +P. ovale*	36.43 + **25.54**	0.00 **+ 28.11**	negative	40.36 + **30.33**	pLDH
*P. falciparum +P. ovale*	**28.40 **+ 37.48	**30.19 **+ 39.60	Pfmx	**30.25 **+ 0.00	HRP-2

Ct-values in bold represent PCR signals obtained for the major *Plasmodium *species. A negative PCR result (Ct-value = 0.00) indicates a missed minor species.

From the 15 patients that demonstrated positive results with PCR on whole blood after starting-up malaria treatment, 11 were detected by PCR on OptiMAL and 7 were positive by PCR on SDFK60 (Table 4). Finally, in none of the negative samples a signal was obtained (Ct = 0) by PCR on both RDT brands (Table 4).

### Comparison of DNA detection by PCR on RDT and antigen detection by RDT

Table 4 shows that PCR on RDT revealed more *Plasmodium *cases than RDT antigen detection. The samples that were missed by antigen detection with the OptiMAL were low parasite density samples (less than 500/μl), except for four missed *P. ovale *samples and one missed *P. malariae *sample that had parasite densities higher than 1,000/μl. The SDFK60 showed visible HRP-2 lines for all samples with *P. falciparum *except the one sample with only gametocytes. Among the non-*falciparum *species that were not visible upon RDT testing by SDFK60 (n = 12), two *P. ovale *and one *P. malariae *samples had densities of more than 1,000/μl (Table 4).

For mixed-infections, antigen detection indicated in three samples with the OptiMAL and in one sample with the SDFK60 RDT the possible presence of more than one *Plasmodium *species by two positive test lines (Table 4 and 5). For the samples after start of treatment, all SDFK60 tests and one OptiMAL test were positive.

### Comparison of Ct-values of PCR on RDT to PCR on whole blood

**Figure 3:**
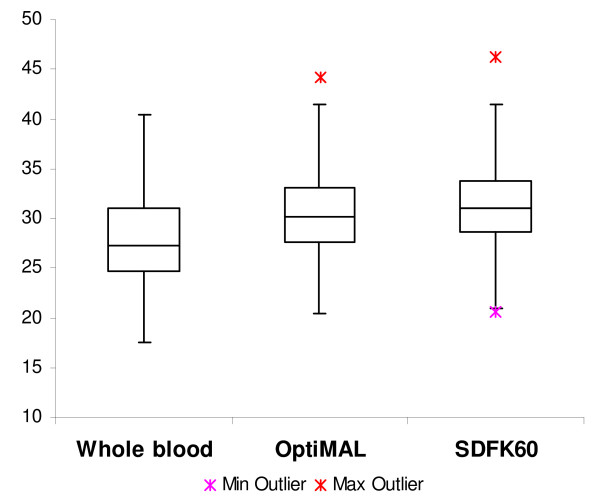
**Boxplot illustration of Ct-values obtained by PCR on whole blood, PCR on OptiMAL and PCR on SDFK60**. The median Ct-value (indicated by the horizontal bar in the box) of PCR on whole blood is significantly (p < 0.01) lower than the median Ct-value of PCR on RDTs. * indicated minimum (min) and maximum (max) outlier.

The correlations (R^2^) between the Ct-values obtained for PCR on whole blood and PCR on RDT were 0.94 for OptiMAL and 0.90 for SDFK60. DNA detection by PCR on RDT generated significantly higher Ct-values than by PCR on whole blood (Figure 3). No significant differences were observed for Ct-values between both RDTs although Ct-values of SDFK60 tended to be higher than of OptiMAL. As illustrated by Figure 3, the median (minimum-maximum) Ct-values were 27.25 (17.48-40.43); 30.24 (20.37-44.14) and 31.05 (20.60-46.30) for PCR on whole blood, OptiMAL and SDFK60 samples respectively. Compared to PCR on whole blood, the mean difference in ∆Ct-values was 2.68 (± 1.29) for PCR on OptiMAL and 3.53 (± 1.33) for PCR on SDFK60.

## Discussion

This study demonstrates the successful recovery of *Plasmodium *DNA from the nitrocellulose component of RDTs by a simple, time saving and low cost DNA elution method. The method proved to be applicable on a wide range of RDT brands. Correct *Plasmodium *species identification by a sensitive real-time PCR assay was possible for the four *Plasmodium *species with varying parasite densities on RDTs stored for weeks to months after routine laboratory diagnosis in a reference setting.

### RDT fragment sampling and DNA extraction

Recently, two reports demonstrated the use of RDTs for the isolation of *Plasmodium *DNA, but the exact RDT fragment sampled was not clearly described. In one report, the filter (called 'blotting') paper that was visibly impregnated with blood after lateral flow migration was selected [15]. On the other hand, Ishengoma *et al *[16] used the 'portion of the strip that contained the sample blotting site'. The present study investigated the DNA extraction from different RDT fragments and studied the sampling method in detail. In contrast to the previous reports, it was demonstrated that best PCR results were obtained from the nitrocellulose component. This finding is quite remarkable and could be explained by two factors. First, it is assumed that the parasite DNA is captured on the nitrocellulose membrane: indeed, such membranes are frequently used for immobilization of nucleic acids in Southern and Northern blotting techniques [17], although the underlying mechanism remains unclear [18]. Second, RDT strips may function as a blood component separator: after blood haemolysis with buffer, the inhibitory blood components are possibly separated from the parasite DNA by the lateral flow. This flow forces for instance haemoglobin to migrate to the filter paper coloring it red/brownish.

In addition, a simple DNA elution method was used yielding high quality DNA which is required for real-time PCR assays [19]. It is known that as little as 0.004% (v/v) blood can inhibit PCR [20]. Inhibitory blood factors can be endogenous (haemoglobin, haem and derivates, lactoferrin, urea, immunoglobulins) or exogenous (heparin) [21-23]. With the Qiagen mini spin columns, removal of inhibitory (blood) components is performed by washing steps while DNA binds onto the silica membrane of the column. The present results show that DNA is even better recovered by the DNA elution method. As inhibitory factors are already separated from the DNA during RDT analysis, the DNA only needs to be eluted from the membrane into the water by heating at 95°C. By contrast, in a previous study, the elution method proved not to be successful for recovery from DNA from thick blood films [10], possibly because material scraped-off from blood films still contains too many inhibitory staining and blood components.

The RDT sampling and DNA elution method is very simple to perform, cheap, fast, and also applicable in field settings. In addition, it is applicable to various RDT brands, mostly by using the nitrocellulose strip. For RDTs with a plastic seal covering the nitrocellulose membrane, the conjugate and/or sample pad were better fragments to sample. For RDT formats of the same brand, the fragment sampling can be performed identical, as demonstrated here for the SDFK50 and SDFK60. Even RDTs on which only 5 μl of blood is applied are excellent sources of DNA as in the present panel the PCR performance was not related to the blood volume applied but more related to the RDT design.

### Real-time PCR on RDT

PCR on RDT can be applied on RDTs archived at room temperature for several weeks or months without effecting *Plasmodium *DNA detection. Here, a very sensitive real-time PCR was used with a short turn-around time [24] compared to the nested PCR format [15,16]. The PCR performed excellent on a well-defined panel of clinical samples in comparison to PCR on whole blood. Species identification was possible in RDTs of all single *Plasmodium *infections with at least one asexual parasite/μl blood. Additionally, PCR on RDT proved to be very sensitive and even able to amplify DNA in RDT samples seeded with low parasite density samples for which no positive test lines were visible by antigen detection. Only for samples of patients after malaria treatment, antigen test lines were more frequently positive than PCR on RDT. It is known that HRP-2 antigens remain longer in circulation after treatment and are thus for a longer period detectable by RDT compared to DNA by PCR in general or parasites by microscopy [25,26].

The lower amount of DNA that can be extracted from RDTs compared to whole blood samples is reflected by higher Ct-values. Despite the lower blood volume, PCR on RDT demonstrated a detection limit of 0.02/μl, which is identical to the detection limit of the same PCR performed on whole blood [9] and compares favorably to the detection limits obtained in previous studies on RDTs [15,16], stained blood films [10,19,27,28] and filter paper cards [29-31]. In contrast, PCR on RDT is less reproducible than by PCR on whole blood [9]. This might be explained by the variability in blood sample distribution over the RDT components during the lateral flow.

### Limitations of the study

A drawback of the DNA elution method is that no extraction control is achievable, for example by the detection of human DNA with the human-beta-globin PCR. No or very weak HBB signals were seen in DNA extracts from RDTs while this was possible for whole blood samples and stained blood films [10,19]. Possibly, human DNA is nearly not released from leucocytes and thus not captured on the nitrocellulose membrane. Another limitation of the present study is that no RDTs from field settings were evaluated. Future prospective studies are needed to investigate the influence of transport and storage conditions of moisture and heat, two factors that could influence RDT stability [26,32].

If PCR on RDT is used as reference method for quality control of RDTs, its high sensitivity might be a drawback. Furthermore, care should be taken for sample contamination during RDT storage, transport and fragment sampling to avoid false-positive PCR results for negative RDTs.

### Applications

PCR on RDT samples opens a window of applications, especially for quality assessment of RDTs. During the past few years, the number of different RDT brands increased enormously, together with the scale of use [33,34]. WHO has already done a comprehensive quality control for RDT product testing [6,7]. Quality control of the RDT performance is a further necessity, especially in endemic regions where malaria diagnosis relies on RDT analysis alone [8,32,35-37]. If standard microscopy is not available, PCR on RDT is the ideal reference due to its ease in sampling and short turn-around time without the additional need for blood collection and storage. As a consequence, this reduces the time of blood collection, the inconvenience of the patients, the administrative work and possible sample identification errors.

For epidemiological studies in remote areas, an additional blood sample is often collected on filter paper cards or DNA cards prior to PCR analysis [23,29,30]. As alternative, stored RDTs which are individually packed in a plastic cassette or card holder can be used. They can easily be stored and shipped to reference or research centers for PCR analysis.

In reference settings, PCR on RDT can be applied for confirmation of malaria infection when the whole blood sample is not available. This frequently happens if patients are already sent home or refuse a second blood collection. Real-time PCR is of added value as it can distinguish between *non-falciparum *species and differentiate between single and mixed infections [9,38]. Another application is post-travel quality control by PCR on for travelers returning home that used a RDT marketed for self-use during their travel with doubtful results.

## Conclusion

This study demonstrates the possibility of using RDTs as specimen for *Plasmodium *species detection and identification by a sensitive and fast real-time PCR method in combination with a simple and cheap DNA elution method. The application of PCR on RDTs of a wide range of brands makes it widely accessible as a tool for quality control in field settings and as confirmation of malaria infection in reference settings.

## Competing interests

The authors declare that they have no competing interests.

## Authors' contributions

LC and JJ designed the study protocol. MB performed extraction and PCR analysis. LC analyzed and interpreted the results. LC, PG, MvE, and JJ drafted the manuscript. LC performed the statistical analysis. All authors read and approved the final manuscript.
